# Supplementary material for: MetaQTL: a package of new computational methods for the meta-analysis of QTL mapping experiments
Source: BMC Bioinformatics. 2007 Feb 8;8:49. doi: 10.1186/1471-2105-8-49 (PMC1808479; doi:10.1186/1471-2105-8-49)
Supplement: Additional File 7 — MetaQTL Package : jar file and tutorial. This Zip archive contains both the MetaQTL JAR file and the files of the tutorial. [file 1471-2105-8-49-S7.zip › org.inra.metaqtl/doc/org/metaqtl/bio/entity/bean/GeneticMapBean.html]

GeneticMapBean


|  |  |  |
| --- | --- | --- |
| **Overview** \| **Package** \| **Class** \| **Use** \| **Tree** \| **Deprecated** \| **Index** \| **Help** | |  |
| **PREV CLASS**   **NEXT CLASS** | **FRAMES**    **NO FRAMES**     **All Classes** |  |
| SUMMARY: NESTED \| FIELD \| CONSTR \| METHOD | DETAIL: FIELD \| CONSTR \| METHOD |  |


---


## org.metaqtl.bio.entity.bean Class GeneticMapBean

```
java.lang.Object
  org.metaqtl.bio.entity.bean.BioEntityBean
      org.metaqtl.bio.entity.bean.GeneticMapBean
```

---

``` public class GeneticMapBean extends BioEntityBean ```

Class Description Here

**Author:**
:   Jean-Baptiste Veyrieras

---

| **Field Summary** | |
| --- | --- |
| `java.util.ArrayList` | `groups` |

**Fields inherited from class org.metaqtl.bio.entity.bean.BioEntityBean**

`name, properties`


**Constructor Summary**

`GeneticMapBean()`


| **Method Summary** | |
| --- | --- |
| `java.util.ArrayList` | `getGroups()` |
| `void` | `setGroups(java.util.ArrayList list)` |

**Methods inherited from class org.metaqtl.bio.entity.bean.BioEntityBean**

`getName, getProperties, setName, setProperties`

**Methods inherited from class java.lang.Object**

`clone, equals, finalize, getClass, hashCode, notify, notifyAll, toString, wait, wait, wait`

**Field Detail**

### groups

```
public java.util.ArrayList groups
```


**Constructor Detail**

### GeneticMapBean

```
public GeneticMapBean()
```


**Method Detail**

### getGroups

```
public java.util.ArrayList getGroups()
```

:   **Returns:**

---


### setGroups

```
public void setGroups(java.util.ArrayList list)
```

:   **Parameters:**: `list` -


---


|  |  |  |
| --- | --- | --- |
| **Overview** \| **Package** \| **Class** \| **Use** \| **Tree** \| **Deprecated** \| **Index** \| **Help** | |  |
| **PREV CLASS**   **NEXT CLASS** | **FRAMES**    **NO FRAMES**     **All Classes** |  |
| SUMMARY: NESTED \| FIELD \| CONSTR \| METHOD | DETAIL: FIELD \| CONSTR \| METHOD |  |


---
